# Total serum IgD from healthy and sick dogs with leishmaniosis

**DOI:** 10.1186/s13071-019-3384-0

**Published:** 2019-03-26

**Authors:** Pamela Martínez-Orellana, Cristina Maristany, Marta Baxarias, Alejandra Álvarez-Fernández, Antonella Baldassarre, Laura Ordeix, Laia Solano-Gallego

**Affiliations:** 1 Departament de Medicina i Cirurgia Animals, Facultat de Veterinària, Universitat Autònoma de Barcelona, Bellaterra, Barcelona, Spain; 2 Università degli Studi di Bari “Aldo Moro”, Bari, Italy; 3 Hospital Clínic Veterinari, Universitat Autònoma de Barcelona, Bellaterra, Spain

**Keywords:** *Leishmania infantum*, Canine leishmaniosis, IFN-γ, IL-10, IgD, PD-1

## Abstract

**Background:**

Canine leishmaniosis (CanL) due to *Leishmania infantum* is characterized by the development of both cellular and humoral immune responses. The dysfunction of T cell-mediated immunity leads to a lack of proliferation of T cells in response to *Leishmania* antigens with the consequence of parasite dissemination that seems to be related to a T cell exhaustion mediated by regulatory B cells expressing immunoglobulin D (IgD). The aim of this study was to determine and compare the total serum IgD in dogs with clinical leishmaniosis and in clinically healthy dogs.

**Results:**

A total of 147 dog sera were studied. All dogs were tested for *L. infantum*-specific antibodies by quantitative ELISA. Interferon-gamma (IFN-γ) production was also determined by sandwich ELISA after blood stimulation with *L. infantum* soluble antigen (LSA) or concanavalin A (ConA). The quantification of total IgD was performed using a human IgD sandwich ELISA quantification set. Dogs were classified in three different groups. Group 1 included 40 clinically healthy non-infected dogs, all serologically negative to *L. infantum*-specific antibodies and non-producers of IFN-γ upon LSA stimulation. Group 2 included 63 clinically healthy infected dogs that were LSA IFN-γ producers (*n* = 61) and/or IFN-γ non-producers (*n* = 2) as well as negative to medium seropositive to *L. infantum* antigen. Finally, Group 3 included 44 dogs with clinical leishmaniosis (IFN-γ producers, *n* = 23; and IFN-γ non-producers, *n* = 21) that were negative to highly positive to *L. infantum*-specific antibodies. No significant differences were observed when the total IgD concentration was compared within groups. Additionally, total IgD of sick IFN-γ producers and IFN-γ non-producers was not significantly different. Finally, total IgD concentration was not statistically related to demographic parameters such as age, sex and breed.

**Conclusions:**

The results of this study demonstrated that there were no differences between groups in total serum IgD. Total serum IgD does not appear to be a marker of disease in CanL.

## Background

Canine leishmaniosis (CanL) is a vector-borne disease caused by the protozoan *Leishmania infantum*, which is mainly transmitted from dog to dog by female sand flies of the genus *Phlebotomus* in Europe [1] and *Lutzomyia* spp. in the New World [2]. The clinical manifestations of this infection are strongly associated with the immune responses developed [3]. After the inoculation of *L. infantum* promastigotes, both innate and adaptive immune responses occur and their balance plays an important role in the outcome of this infection [4]. The adaptive response includes the activation of B and T lymphocytes that are always present during *Leishmania* infection. B lymphocytes induce a humoral response with exacerbated antibodies production that seems to be related to a T cell exhaustion resulting in failure to control the infection, meanwhile T lymphocytes play an important role in the development of a cellular response. Dogs with severe disease have a very strong humoral response together with a reduction or absence of the T cell-mediated immunity [1].

*Leishmania infantum* infection does not always indicate the development of a clinical disease [5]. This infection can manifest as a chronic subclinical infection, self-limited disease or a severe fatal disease. Dogs that develop clinical disease are divided into four clinical stages according to clinical signs, clinicopathological abnormalities and the levels of specific antibodies [6, 7]. Moreover, dogs with mild to moderate clinical stages present higher interferon-gamma (IFN-γ) production, low parasite load and antibody response when compared to the more severe stages [8, 9].

Interestingly, some breeds appear to be more likely to control infection while other breeds are more susceptible. For example, the Ibizan Hound rarely develops clinical disease [10] in contrast breeds such as bulldogs, dobermanns [11], German shepherds, rottweilers, boxers or cocker spaniels commonly develop clinical leishmaniosis [1, 12]. Other factors that might contribute in the progression of clinical leishmaniosis are sex, nutritional state, virulence of *Leishmania*, parasitic load, co-infections, other non-infectious debilitating diseases, stress and immunosuppressive treatments [6, 13–15].

As mentioned above, dogs with moderate to severe clinical leishmaniosis present a reduced or absent parasite-specific T cell response. The cellular basis and mechanisms for the development of antigen-specific T cell unresponsiveness in CanL are not fully understood. However, a study demonstrated a concurrent CD4^+^ and CD8^+^ T cell exhaustion in dogs with clinical leishmaniosis by a surface overexpression of programmed death 1 protein (PD-1) that inhibited their function and co-induced a low production of IFN-γ and an increase of interleukin-10 (IL-10) production. Blockade of IL-10 and PD-1 recovered both CD4^+^ and CD8^+^ T cell proliferation and CD4^+^ IFN-γ production but also reduced IL-10 production [16].

The activation of B lymphocytes and plasmatic cells leads to a massive anti-*Leishmania* antibodies production, mainly immunoglobulin G (IgG), but also other immunoglobulins like IgM, IgE and IgA [17–19]. The levels of *L. infantum*-specific immunoglobulin isotypes are higher in sick dogs than in clinically healthy infected dogs [18–20]. Immunoglobulin D (IgD) was first discovered in 1965 as a human myeloma protein [21, 22] but then was also detected in mice, rats, rabbits and guinea pigs [23]. In 1994, a study identified an IgD-like molecule in dogs [24]. IgD is a surface marker for naïve B cells before isotype switching and for cells with B cell regulatory dysfunction [15, 20]. IgD secretion may be a marker of a regulatory phenotype during any chronic infection, but is unlikely to be antigen- or disease-specific. As mentioned before, normal canine B cells co-express IgM and IgD [17, 22] and produce IL-10 but *Leishmania-*infected sick dogs have high levels of IgM^+^/IgD^+^ and IgM^-^/IgD^+^ and low levels of IgM^+^/IgD^-^ [17]. The population of IgD^+^ B cells increases with disease progression that conduces to IL-10 release and an increase of the inhibitory receptor PD-1, leading to T cell function suppression and to cellular exhaustion. Furthermore, these cells induce other B and T lymphocytes to produce IL-10 and supress IFN-γ through PD-1 [17]. In addition, a higher concentration of total serum IgD in *L. infantum* infected sick dogs when compared with healthy non-infected dogs was demonstrated [17]. However, a very limited number of dogs were investigated [17]. We hypothesized that dogs with moderate to severe disease with a high antibody response and absence of production of *L. infantum* specific IFN-γ in *ex vivo* blood [8] might have a higher concentration of serum total IgD when compared with sick dogs with mild to moderate disease but able to produce parasite specific IFN-γ and when compared with clinically healthy dogs as previously described [17]. Therefore, the aim of this study was to determine total serum IgD in dogs with clinical leishmaniosis at the time of diagnosis (IFN-γ producers and non-producers) and to compare with clinically healthy non-infected and infected dogs and also to correlate with clinical and immunological parameters.

## Methods

### Dogs and sampling

A total of 147 canine archived serum samples were retrospectively used. The study was conducted during 2015 and 2017. The dogs were classified into three different groups. Group 1 included 40 control non-infected clinically healthy dogs that were negative to *L. infantum*-specific antibodies and parasite-specific IFN-γ non-producers. Group 2 included samples collected from 63 apparently clinically healthy infected dogs that were negative, low- or medium-positive to *L. infantum*-specific antibodies and the majority were IFN-γ producers (*n* = 61) with the exception of two dogs that were IFN-γ non-producers but low seropositive. Samples of both control groups were collected in different locations: Fundació Hospital Clínic Veterinari (Bellaterra, Barcelona) (Group 1: 10 dogs; Group 2: 6 dogs), island of Mallorca (Balearic islands, Spain) (Group 1: 10 dogs; Group 2: 48 dogs), Asturias (Spain) (Group 1: 17 dogs; Group 2: 4 dogs) and Facoltà de Veterinaria of Università degli Studi di Bari “Aldo Moro” (Bari, Italy) (Group 1: 3 dogs; Group 2: 5 dogs).

Group 3 was composed of 44 dogs with clinical leishmaniosis at the time of diagnosis that were negative to highly positive to *L. infantum*-specific antibodies and were further classified as IFN-γ producers or IFN-γ non-producers. Additionally, sick dogs were also studied depending on their clinical staging (I, IIa, IIb, III, IV) according to LeishVet guidelines [6]. The samples were taken at diagnosis in Fundació Hospital Clínic Veterinari (Bellaterra, Barcelona) (31 dogs), Hospital Ars Veterinària (Barcelona) (8 dogs), Consultori Montsant (Falset, Tarragona) (4 dogs) and Facoltà de Veterinaria of Università degli Studi di Bari “Aldo Moro” (Bari, Italy) (1 dog).

All serum samples of all dogs studied were treated equally and stored at -80 °C. *Leishmania infantum*-specific antibodies were measured by endpoint ELISA in all dogs studied as previously described [25]. Determination of IFN-γ production was measured by sandwich ELISA after heparin whole blood stimulation with *L. infantum* soluble antigen (LSA) and concanavalin A (ConA). Dogs were considered producers when IFN-γ was detectable after subtracting medium alone [8].

### Detection of total serum IgD by sandwich ELISA

Quantification of canine IgD was performed according to the human IgD ELISA Quantitation Set from Bethyl Laboratories® (Montgomery, USA) with slightly modifications from Schaut et al. [17].

Briefly, 100 µl of coating antibody at a dilution of 1:5000 in carbonate-bicarbonate buffer was added to each well and incubated at room temperature for 1 h. Then, five washes were performed with 200 µl of phosphate-buffered saline (PBS) with 0.01% of Tween-20. Then, 200 µl of blocking solution composed of PBS and 1% of bovine serum albumin (BSA) was added to each well and incubated for 30 min at room temperature. After washing five times in PBS 0.01% Tween-20, 100 µl of standards and undiluted serum samples was added per duplicate and incubated for 1 h at room temperature. Then, five PBS 0.01% Tween-20 washes were repeated and 100 µl of diluted horseradish peroxidase (HRP) detection antibody diluted at 1:20,000 in PBS was added and incubated for 1 h at room temperature. After washing five times in PBS 0.01% Tween-20, 100 µl of 3,3′,5,5′-tetramethylbenzidine (TMB) substrate solution was added to each well and incubated for 15 min. Then, 100 µl of stop solution (HCL 5M) was added. Finally, the absorbance was measured using an automatic micro ELISA reader Anthos 2020 (Biochrom, Cambridge, UK) at 450 nm [17].

Standards were prepared by mixing human reference serum included in the IgD ELISA kit with buffer diluent (blocking buffer plus 0.01% of Tween-20). Each plate had a total of eight standards per duplicate with concentrations between 3.9 and 500 ng/ml. Quantification was performed using a four-parameter logistic curve in My Assays® (https://www.myassays.com/). Furthermore, plates were repeated when the *R*^2^-value of the standard curve was below 0.98.

### Statistical analysis

A descriptive study of signalment (sex, age and breed), total serum IgD concentration, clinical status, level of *L. infantum* antibodies and IFN-γ production was performed. Chi-square and Fisher’s exact test were used to compare proportions of categorical data (sex, age, breed, seropositivity to *L. infantum* and IFN-γ producers or non-producers) by dog groups and clinical stages. Dogs were classified as young if they were equal to or less than 18 months of age or as adults if they were more than 18 months of age, in order to change the numeric data to categorical data. Comparisons of IgD concentration between signalment binomial data (sex, age, breed, seropositivity to *L. infantum* and IFN-γ producer or non-producer) and between dog groups and clinical stages for quantitative variables (parasite specific antibody levels, IgD concentration and LSA and ConA IFN-γ production) were performed using the non-parametric Mann-Whitney U-test. Association between parasite-specific antibody response, IgD concentration and age, LSA and ConA IFN- γ production was assessed with Spearman’s correlation.

A *P*-value of < 0.05 was considered statistically significant. The statistical analysis was performed using the moments and mass packages [26, 27] for the software R i386 v.3.4.2 [28] and Deducer v.1.8-4 [29] for Windows. Graphics were built using GraphPad Prism 8 (GraphPad Software, San Diego, Canada).

## Results

### Clinical findings, *L. infantum* antibodie*s* and IFN-γ production

In the control non-infected Group 1, there were a total of 40 dogs. The average age at recruitment was 4.2 years with a range from 4 months to 13 years. Twenty were female, 15 were male and five were not defined due to lack of information. Mixed breeds and 15 pure breeds were represented. All 40 dogs were seronegative to *L. infantum-*specific antibodies.

**Fig. 1:**
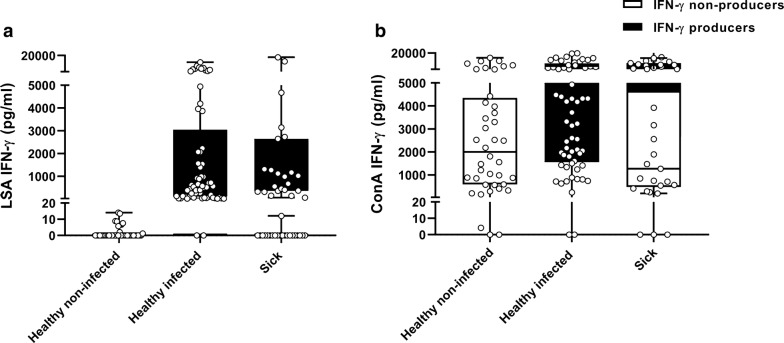
**a** Specific *L. infantum* IFN-γ concentration based on IFN-γ producers and IFN-γ non-producers in healthy non-infected, healthy infected and sick dogs. **b** IFN-γ concentration after ConA stimulation based on IFN-γ producers and IFN-γ non-producers in healthy non-infected, healthy infected and sick dogs. White circles represent individual data of each dog, boxes represent minimum and maximum values of quartiles, the bands inside represent the second quartile (median). Statistical results of LSA IFN-γ: Group 1 (healthy non-infected) < Group 2 (healthy infected) (Mann-Whitney U-test: *Z* = -8.31, *P* < 0.0001), Group 1 (healthy non-infected) < Group 3 (sick) (Mann-Whitney U-test: *Z* = -3.81, *P* = 0.0001), Group 2 (healthy infected) < Group 3 (sick) (Mann-Whitney U-test: *Z* = -3.28, *P* = 0.001). ConA IFN-γ: Group 1(healthy non-infected) < Group 2 (healthy infected) (Mann-Whitney U-test: *Z* = -2.67, *P* = 0.008). *Abbreviations*: LSA, *Leishmania* soluble antigen; ConA, concanavalin A; IFN-γ, interferon gamma

Control healthy non-infected dogs were all IFN-γ non-producers with lower production after LSA (Mann-Whitney U-test: *Z* = -8.31, *P* < 0.0001) and ConA (Mann-Whitney U-test: *Z* = -2.67, *P* = 0.008) stimulation when compared with control healthy infected IFN-γ producers (Fig. 1a, b).

**Fig. 2:**
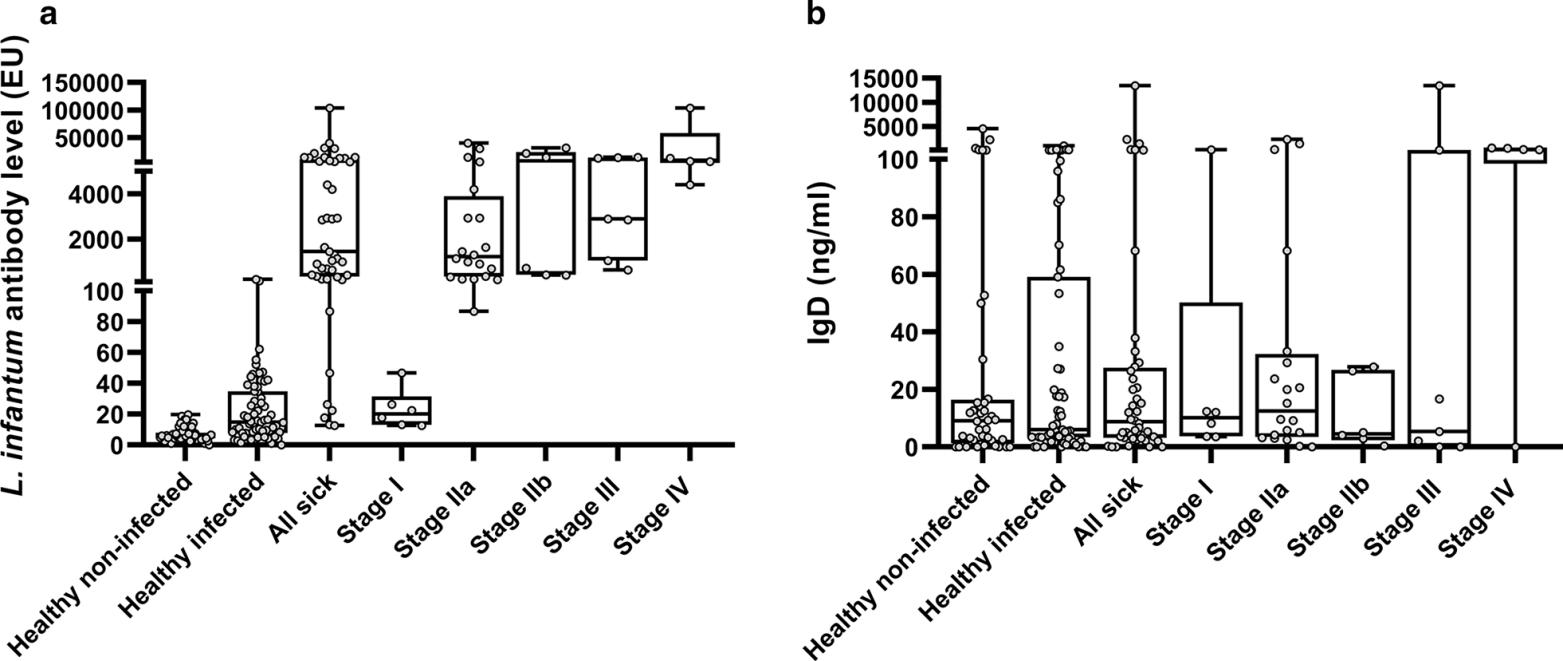
**a** Specific *L. infantum* antibodies levels in healthy non-infected, healthy infected and sick dogs classified based on LeishVet clinical staging. **b** Total serum IgD concentrations in healthy non-infected, healthy infected and sick dogs classified based on LeishVet clinical staging. White circles represent individual data of each animal, boxes represent minimum and maximum values of quartiles, the bands inside represent the second quartile (median). Statistical results of *L. infantum* specific antibodies by ELISA: Group 1 (healthy non-infected) < Group 2 (healthy infected) (Mann-Whitney U-test: *Z* = -4.46, *P* < 0.0001), Group 1 (healthy non-infected) < Group 3 (sick) (Mann-Whitney U-test: *Z* = −9.59, *P* < 0.0001), Group 2 (healthy infected) < Group 3 (sick) (Mann-Whitney U-test: *Z* = -7.84, *P* < 0.0001), mild stages (I and IIa) < severe stages (IIb, III and IV) (Mann-Whitney U-test: *Z* = -3.07, *P* = 0.002), stage I < stage IIa (Mann-Whitney U-test: *Z* = -4.45, *P* < 0.0001), stage I < stage IIb (Mann-Whitney U-test: *Z* = -3.07, *P* = 0.002), stage I < Stage III (Mann-Whitney U-test: *Z* = -3.25, *P* = 0.001), stage I < stage IV (Mann-Whitney U-test: *Z* = -2.85, *P* = 0.004), stage IIa < stage IV (Mann-Whitney U-test: *Z* = -2.59, *P* = 0.01)

A total of 63 apparently clinically healthy infected dogs were included in Group 2. The average age was 4.4 years with a range from 4 months to 10 years. Thirty-six were female, 16 were male and 11 were not defined due to a lack of information. Mixed breeds and 11 pure breeds were represented. The results of the antibody levels of Group 2 are shown in Fig. 2a. Fifteen out of 63 dogs presented low to medium levels of *L. infantum* antibodies and the remaining dogs were seronegative. The proportion of IFN-γ producers and IFN-γ non-producers on this group of healthy infected dogs was 96.8% (*n* = 61) and 3.1% (*n* = 2), respectively (Fig. 1a). The two IFN-γ non-producers were low seropositive.

In the group of sick animals (Group 3) there were a total of 44 dogs with an average age of 4.7 years with a range from 5 months to 17 years. Nineteen were female, 24 were male and sex was not identified in one dog. Mixed breeds and 19 pure breeds were represented.

Dogs from Group 3 were classified in four clinical stages. *Leishmania-infantum* antibody levels and statistically significant differences are described in Fig. 2a. Sick dogs (*n* = 44) presented statistically significant higher levels of antibodies against *L. infantum* (Mann-Whitney U-test: *Z* = -8.9, *P* < 0.0001) when compared with control dogs (Groups 1 and 2, *n* = 103). A tendency of higher *L. infantum* antibody levels was found in IFN-γ non-producers when compared with IFN-γ producers sick dogs (Mann-Whitney U-test: *Z* = -1.91, *P* = 0.056). Higher concentration of IFN-γ after LSA (Mann-Whitney U-test: *Z* = -5.96, *P* < 0.0001) and ConA (Mann-Whitney U-test: *Z* = -2.53, *P* = 0.01) was found in sick dogs IFN-γ producers when compared with IFN-γ non-producers (Fig. 1a, b).

IFN-γ producer sick dogs presented significantly higher *L. infantum* antibody levels (Mann-Whitney U-test: *Z* =-7.94, *P* < 0.0001), and IFN-γ production after LSA (Mann-Whitney U-test: *Z* = -6.95, *P* < 0.0001) and ConA (Mann-Whitney U-test: *Z* = -2.57, *P* = 0.01) when compared with IFN-γ non-producer healthy dogs (Fig. 1a, b).

Differences between sex, age and breed were not found between all the three groups studied. Furthermore, when all dogs studied were divided into seropositive or seronegative, no differences were found regarding signalment (sex and breed). However, a statistically significant difference was found between age and serology with young dogs more likely to be seronegative (Chi-square test: *χ*^2^ = 9.37, *df* = 1, *P* = 0.002). Nevertheless, the Mann-Whitney U-test revealed no significant differences for age between seronegative and seropositive dogs (*Z* = -1.23, *P* = 0.22).

### IgD concentration

**Fig. 3:**
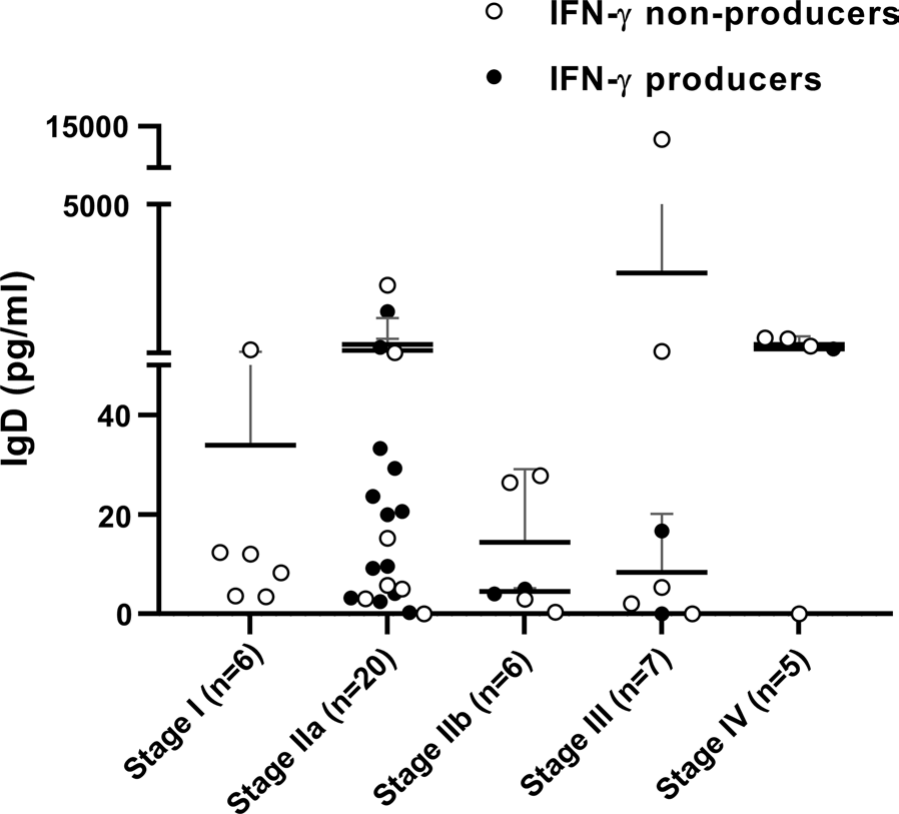
Total serum IgD concentrations in IFN-γ producers and IFN-γ non-producers sick dogs classified based on LeishVet clinical staging. Circles represent individual data of each dog, the horizontal line represents the mean and the vertical lines represent the standard deviation. No statistical differences were observed within LeishVet clinical stages. *Abbreviations*: LSA, *Leishmania* soluble antigen; ConA, concanavalin A; IFN-γ, interferon gamma

The results of IgD concentration for each group are shown in Figs. 2b, 3. IgD concentration between groups did not show any significant differences when sick dogs were compared with control healthy non-infected and healthy infected dogs as well when both groups of control animals were compared (Fig. 2b). Moreover, when IgD concentrations were compared between sick dogs IFN-γ producers and sick dogs IFN-γ non-producers no significant differences were detected (Fig. 3). In addition, no statistical differences were found when IgD concentrations were compared between the different LeishVet clinical stages (Fig. 3). Furthermore, statistically significant differences of total IgD concentrations were also not found when sick dogs were grouped based on LeishVet clinical stages (stages I-IIa *versus* stages IIb-III-IV).

IgD concentration based on dichotomous parameters such as signalment (sex, age and breed), *L. infantum* serology and IFN-γ production are summarized in Table 1.

**Table 1:** IgD concentration based on dichotomous parameters such as signalment (sex, age and breed), *L. infantum* serology and IFN-γ production

Variable	Group	IgD concentration (ng/ml)	Mann–Whitney test		
		Mean ± SD	*U*-value	*Z-*value	*P*-value
Sex	Male (*n* = 54)	391.33 ± 1873.22	1922	-0.37	0.7136
	Female (*n* = 74)	41.4 ± 124.27			
Age	≤ 18 months (*n* = 35)	14.85 ± 31.46	1960	-1.89	0.0583
	> 18 months (*n* = 92)	259 ± 1442.28			
Breed	Purebred (*n* = 108)	177.81 ± 1302.62	1588	-0.44	0.6632
	Mixed breed (*n* = 31)	168.69 ± 568.43			
*L. infantum* serology	Positive (*n* = 54)	351.08 ± 1855.6	2383	-0.51	0.6068
	Negative (*n* = 93)	115.76 ± 534.48			
IFN-γ production	Producer (*n* = 63)	62.32 ± 194.48	2599	-0.18	0.8555
	Non-producer (*n* = 84)	388.71 ± 1812.55			

*Abbreviations*: IgD, immunoglobulin D; IFN-γ, interferon gamma; n, number of dogs; SD, standard deviation

### Correlations

A positive correlation between *L. infantum* antibodies, age and IFN-γ production was found when all dogs from the three groups studied (*n* = 147) were analysed (Table 2). However, no significant correlation of the antibody response to *L. infantum* antigen or IFN-γ production and IgD concentration was encountered.

**Table 2:** Spearman’s correlation between age, IgD concentration, LSA IFN-γ concentration and serology by groups studied

	Spearman’s *r*_*s*_ (*P*-value)	
	IgD concentration (ng/ml)	*L. infantum* antibodies (EU)
All dogs (*n* = 147)		
Age (months)	0.0220 (0.8096)	0.2024 (0.0254)*
IgD concentration (ng/ml)	1	0.0307 (0.7120)
*L. infantum* antibodies (EU)	0.0307 (0.7120)	1
LSA IFN-γ concentration (pg/ml)	0.0438 (0.5983)	0.1742 (0.0348)*
All healthy dogs (*n* = 103)		
Age (months)	0.1664 (0.1401)	0.0713 (0.5300)
IgD concentration (ng/ml)	1	0.0067 (0.9468)
*L. infantum* antibodies (EU)	0.0067 (0.9468)	1
LSA IFN-γ concentration (pg/ml)	0.0481 (0.6297)	0.4872 (< 0.0001)*
Sick dogs (*n* = 44)		
Age (months)	-0.2665 (0.0881)	0.1104 (0.4862)
IgD concentration (ng/ml)	1	0.0297 (0.8483)
*L. infantum* antibodies (EU)	0.0297 (0.8483)	1
LSA IFN-γ concentration (pg/ml)	0.0549 (0.7235)	-0.3233 (0.0323)*
Control healthy dogs (non-infected) (*n* = 40)		
Age (months)	0.1206 (0.5038)	0.0609 (0.7366)
IgD concentration (ng/ml)	1	-0.1898 (0.2408)
*L. infantum* antibodies (EU)	-0.1898 (0.2408)	1
LSA IFN-γ concentration (pg/ml)	-0.0104 (0.9493)	-0.2315 (0.1506)
Control healthy dogs (infected) (*n* = 63)		
Age (months)	0.2145 (0.1476)	0.0735 (0.6235)
IgD concentration (ng/ml)	1	0.0692 (0.5899)
*L. infantum* antibodies (EU)	0.0692 (0.5899)	1
LSA IFN-γ concentration (pg/ml)	0.0124 (0.9232)	0.4212 (0.0006)*

*Statistically significant *P*-values

*Abbreviations*: LSA, *Leishmania* soluble antigen; ConA, concanavalin A; IFN-γ, interferon gamma; IgD, immunoglobulin D; EU, ELISA units

A similar positive correlation was found between *L. infantum* antibodies and IFN-γ production when the control healthy dogs included in Groups 1 and 2 (*n* = 103) and also when only Group 2 (healthy infected) were analysed (Table 2). No correlation was observed with any of the parameters studied (*L. infantum* antibodies or IFN-γ production or age) and IgD concentration in Group 1 (healthy non-infected).

Finally, sick dogs (Group 3; *n* = 44) were studied separately and a negative correlation was found between *L. infantum* antibodies and LSA IFN-γ production (Table 2). Once again, no correlation was found with any of the parameters studied (*L. infantum* antibodies or IFN-γ production or age) and IgD concentration.

## Discussion

IgD is expressed on the surface of B cells and is usually accompanied by IgM [22]. Knowledge about the role of IgD in dogs has progressed little since the discovery of the molecule IgD-like in 1994 [24].

The progression of clinical CanL has been related to T cell exhaustion by an overexpression of PD-1, which increases the levels of IL-10 and decreases the production of IFN-γ [16]. A previous study demonstrated that all dogs have regulatory B cells expressing IgD and producing IL-10, but dogs with clinical leishmaniosis tend to have higher levels of B cells expressing IgD, leading to an increase of PD-1 and IL-10 and a progression of the disease [17]. In addition, the same group showed a significant higher concentration of total serum IgD in sick dogs (*n* = 16) when compared with healthy non-infected dogs (*n* = 10) [17]. In the present study, we aimed to investigate and compare the total serum IgD in clinically healthy and sick dogs. Unfortunately, results presented here did not show a significant higher concentration of serum total IgD in all sick dogs when compared with all healthy dogs (non-infected and infected) or when compared healthy non-infected dogs or healthy infected dogs alone. Nevertheless, an increase of total IgD concentration in all sick dogs was found when compared with both groups of healthy infected and non-infected dogs (see Fig. 2b). However, those differences were not statistically significant. For this reason, results presented in this study do not fully confirm previous work [17] but suggest a similar tendency of high concentration of total IgD in dogs with clinical leishmaniosis.

Furthermore, when control groups were compared, a higher but not significant level of total IgD was found in clinically healthy non-infected dogs when compared with clinically healthy infected dogs (Fig. 2b). Conversely, Schaut et al. [17] observed an opposite trend, although this difference was also not significant. Our findings might suggest that total IgD does not appear to increase in healthy dogs infected with *L. infantum*.

Since human IgD was discovered [23], it has been identified in many other diseases and infections such as the immunodeficiency virus [30], leprosy [31], tuberculosis [32], malaria [33, 34], hepatitis [35] and patients suffering from hyper-IgD and periodic fever syndrome [36]. The production of pathogen-specific IgD in response to infection [22, 23] as it binds to pathogenic microorganisms and their virulence factors has been associated to *Streptococcus* streptolysin O [37], *Moraxella catarrhalis* [38] and *Haemophilus influenzae* adhesion [39] which is called *Moraxella* IgD binding protein/hemagglutinin. In human patients, IgD is increased in many pathologic problems, including infections, autoimmune diseases, immunodeficiencies and allergies [23]. It is important to highlight that, so far, there is limited information regarding IgD in dogs and its release under clinicopathologic conditions. To the best of our knowledge, IgD has only been investigated in one study in CanL [17] other than the present study. It is likely that the total serum IgD might increase in other clinical conditions such as other infections in dogs as previously reported in humans [23]. Therefore, the present results might be influenced by the presence of concomitant diseases (such as atopy or other allergies) or infections, which would increase the total serum IgD concentration without being related to *L. infantum* infection. Unfortunately, in the present study it was not possible to investigate the possibility of concomitant infections or levels of IgE against different allergens present in all the dogs studied. In addition, it remains unknown if the concentration of total serum IgD might be different depending on provenience, age, sex, breed or clinical background. However, in the present study, the total serum IgD concentration appears not to be different based on sex and breed while age might have influenced this immunoglobulin. In this study, young dogs presented a trend of lower concentration of serum total IgD when compared with adult dogs. However, Spearman’s correlation did not support this finding. Studies at least in other canine clinical conditions would be of interest.

In the present study and based on the findings previously described [17], we hypothesized that dogs with a pattern of moderate to severe disease, high antibody response and absence of specific *L. infantum* IFN-γ response might have higher IgD serum concentration when compared with sick dogs with mild to moderate disease, low parasite load and parasite-specific IFN-γ response [8]. Unfortunately, we did not find significant differences on IgD concentrations between sick dogs *L. infantum* IFN-γ producers and IFN-γ non-producers. In addition, significant differences of total IgD concentrations were also not encountered when sick dogs were grouped based on LeishVet clinical stages (stages I-IIa *versus* stages IIb-III-IV). Therefore, serum total IgD does not seem to be a useful marker of disease severity in clinical leishmaniosis in dogs. In contrast, our findings showed *L. infantum* specific IFN-γ in *ex vivo* blood and *L. infantum* specific antibody levels are useful markers to assess disease severity in agreement with previous reports [8, 9]. IFN-γ producer dogs are strongly associated with mild to moderate clinical staging (stages I and IIa) while IFN-γ non-producer dogs have been associated with more severe clinical stages (stages IIb, III and IV). On the contrary to the clinical analysis performed here, the previous study compared the total IgD measured in sick dogs with leishmaniosis, based on physical examination and classification of clinical staging was not performed, which means that sick dogs were classified as PCR positive with high serological levels as symptomatic [17]. In addition, the IFN-γ concentration after stimulation with *Leishmania* antigen and its relation with IgD levels was not described [17].

Limitations of this study include its retrospective nature that hindered having all clinical data of the dogs; for example, the existence of concomitant infections or the effect of chronic clinical conditions such as atopy or other allergies. The ELISA employed was for the detection of human IgD and it seemed to work well as demonstrated here and previously [17]. However, the use of proper canine antibodies might increase the detection and performance of canine IgD. Furthermore, it is important to remark that it is also likely that the present study lacks statistical power to detect subtle differences between groups. Ultimately, the lack of tools to understand how *Leishmania-*specific IgD behaves during infection and disease hampers a greater interest in the presented data as many confounding factors associated with IgD evaluation exist.

## Conclusions

This study demonstrates that there is no clear increase of total serum IgD in dogs with clinical leishmaniosis when compared with healthy dogs, although a trend was found. Interestingly, higher but not statistically significant levels of total serum IgD were found in clinically healthy non-infected dogs when compared with infected dogs. Additionally, higher but not significant total serum IgD was observed in sick dogs classified as IFN-γ non-producers comparing to IFN-γ poducers. Therefore, total serum IgD does not seem to be a useful marker for establishing disease in clinical leishmaniosis in dogs.

## Abbreviations

BSA: bovine serum albumin
CD: cluster of differentiation
CanL: canine leishmaniosis
ConA: concanavalin A
ELISA: enzyme-linked immunosorbent assay
EU: ELISA units
HCL: hydrogen chloride
HRP: horseradish peroxidase
IFN-γ: interferon-gamma
IgA: immunoglobulin A
IgD: immunoglobulin D
IgE: immunoglobulin E
IgG: immunoglobulin G
IgM: immunoglobulin M
IL-10: interleukin-10
LSA: *Leishmania infantum* soluble antigen
PBS: phosphate-buffered saline
PD-1: programmed death 1 protein
SD: standard deviation
TMB: 3,3′,5,5′-tetramethylbenzidine
